# The roles of mitoferrin-2 in the process of arsenic trioxide-induced cell damage in human gliomas

**DOI:** 10.1186/s40001-014-0049-5

**Published:** 2014-09-26

**Authors:** Chunlei Wang, Xiaofeng Chen, Huichao Zou, Xin Chen, Yaohua Liu, Shiguang Zhao

**Affiliations:** Department of Neurosurgery, The First Affiliated Hospital of Harbin Medical University, Harbin, People’s Republic of China

**Keywords:** Glioma, Mitoferrin-2, Apoptosis, Oxidative stress, Arsenic trioxide

## Abstract

**Background:**

Among glioma treatment strategies, arsenic trioxide (As_2_O_3_) has shown efficacy as a therapeutic agent against human gliomas. However, the exact antitumor mechanism of action of As_2_O_3_ is still unclear. Mitochondria are considered to be the major source of intracellular reactive oxygen species (ROS), which are known to be associated with As_2_O_3_-induced cell damage. Therefore, we investigated whether mitoferrin-2, a mitochondrial iron uptake transporter, participates in As_2_O_3_-induced cell killing in human gliomas.

**Methods:**

Human glioma cell lines were used to explore the mechanism of As_2_O_3_’s antitumor effects. First, expression of mitoferrin-2 was analyzed in glioma cells that were pretreated with As_2_O_3._ Changes in ROS production and apoptosis were assessed. Furthermore, cell viability was assessed by 3-(4,5-dimethylthiazol-2-yl)-2,5-diphenyltetrazolium bromide (MTT).

**Results:**

In the present study we found that As_2_O_3_ induced ROS production and apoptosis in glioma cells. In addition, gene expression of mitoferrin-2, a mitochondrial iron uptake transporter, was increased 4 to 5 fold after exposure to As_2_O_3_ (5 μM) for 48 hours. Furthermore, apoptosis and cytotoxicity induced by As_2_O_3_ in glioma cells were decreased after silencing the *mitoferrin-2* gene.

**Conclusions:**

Our findings indicated that mitoferrin-2 participates in mitochondrial ROS-dependent mechanisms underlying As_2_O_3_-mediated damage in glioma cells.

## Background

Glioma is a serious life-threatening disease, having the characteristics of invasiveness and a dismal prognosis. Chemotherapy and/or radiotherapy are the main approaches for glioma treatment after surgery. As_2_O_3_ as an antitumor agent has shown efficacy in treating glioma in laboratory tests [1,2]. Additionally, As_2_O_3_ has potential as a mitochondrial toxin that can induce reactive oxygen species (ROS) production; these mainly originate in the intracellular mitochondrial respiratory chain and further toxic byproducts subsequently produced could lead to mitochondrial damage [3]. In addition, excessive ROS production acts as key mediator of the apoptotic-signaling pathway. However, the detailed mechanism of As_2_O_3_-induced excessive ROS production remains unclear.

Among numerous possible factors, such as altering the activity of enzymes, kinases, phosphatases and transcription factors [4], iron has been demonstrated in previous studies [5–9] to play an important role during ROS production in the mitochondrion. Because of the character of its redox properties, iron can catalyze the production of ROS, which are highly toxic [10], and increase the redox reaction [11].

Two main forms of iron exist in the cell: non-chelatable iron and chelatable iron. Lysosomes store amounts of chelatable iron [12], which promotes oxidative stress by catalyzing the Fenton reaction and produces highly reactive hydroxyl radicals. Excessive reactive hydroxyl radicals could damage DNA, proteins, and membranes. The concentration of cytosolic chelatable iron is low under normal conditions, but, in pathological conditions, chelatable iron released from lysosomes can increase cytosolic iron concentration [12]. Additionally, thelysosome-damaging ability of arsenite has been demonstrated [13] and it is also known that excessive mitochondrial ROS production requires more iron to cross the mitochondrial membrane. Therefore, we hypothesized that the mitochondrial iron transporter may participate in As_2_O_3_-induced excessive ROS production.

Mitochondria are considered to be the major source of intracellular ROS, which are knowingly associated with retrograde signaling and affect many cellular functions [14–16]. Mitoferrin, a mitochondrial protein, has been reported to mediate ferrous iron transport across the mitochondrial inner membrane [11,17,18]. Mitoferrin has two isoforms: mitoferrin-1 and mitoferrin-2, which are localized on the inner mitochondrial membrane and function as a requisite importer of iron for mitochondrial heme and iron-sulfur cluster (ISC) in erythroblasts [11]*.* Mitoferrin-1 is mainly distributed in erythroid cells with low levels in other tissues, whereas mitoferrin-2 is ubiquitously distributed [19]. In non-erythroid cells, mitoferrin-2 possibly functions to maintain the levels of cellular mitochondrial iron [11,19]. Previous research has reported that mitoferrin-2 transmits ferrous iron from cytoplasm to mitochondria. Additionally high mitoferrin-2-expressing cells showed higher rates of mitochondrial ferrous iron uptake compared with low mitoferrin-2-expressing cells [20]. Therefore, the possibility that the mitoferrin-2 transporter participates in As_2_O_3_-induced apoptosis in glioma should be considered.

In this study, our aim is to investigate whether mitoferrin-2 participates in the cytotoxic effect of As_2_O_3_ in human glioma and mediates the production of ROS.

## Methods

### Source of reagents

As_2_O_3_ compound was obtained from the Department of Pharmacy, the First Affiliated Hospital of Harbin Medical University (Harbin, China), and fresh dilutions with DMEM were used in each experiment. Mitoferrin-2 siRNA was designed and purchased from GenePharma (Shanghai, China). Annexin V-FITC-PI apoptosis detection kit (Baosea Biotechnology Co., Beijing, China) was used for detection of apoptosis by flow cytometry. For detection of ROS activity, 2′,7′-dichlorofluorescin diacetate (Sigma-Aldrich, St. Louis, MO, USA) was used. Reverse transcriptase RT kit and real time PCR kit (Takara Biotechnology Co., Shiga, Japan) were used for the semiquantitation of mitoferrin-2 mRNA levels. Dimethylsulfoxide (DMSO) and 3-(4,5-dimethylthiazol-2-yl)-2,5-diphenyltetrazolium bromide (MTT) (Sigma-Aldrich, St. Louis, MO, USA) were used for detection of cell viability.

### Cell culture

Human glioma cell lines, U87MG and T98G were cultured in DMEM(Hyclone, Logan, UT, USA) supplemented with 10% FBS(Hyclone, Logan, UT, USA) at 37°C in a humidified CO_2_ incubator, and 1% penicillin-streptomycin. The cells were passaged twice weekly, and once they were nearly confluent, they were released with 0.25% trypsin-ethylenediaminetetraacetic acid (EDTA) [21].

### RNA interference studies

The siRNA specific sequences of human mitoferrin-2 transporter were designed according to standard procedures, and obtained from Shanghai GenePharma (Shanghai, China). The sequences were:

5′-GGCAACAUUACUUCAUGAUTT-3′ and 5′-AUCAUGAAGUAAUGUUGCCTT-3′; and the negative control sequences were:

5′-UUCUCCGAACGUGUCACGUTT-3′ and 5′-ACGUGACACGUUCGGAGAATT-3′. Glioma cells were transfected with 40 pmol of siRNA duplex or negative groups and exposed to 5 μM As_2_O_3_ for 48 hours.

### Quantitative realtimePCR (QRT-PCR)

Total RNA was isolated from glioma cells using Trizol reagent (Invitrogen, California, USA). It was then reverse-transcribed to cDNA with random primers using a reverse transcriptase RT kit (Takara Biotechnology Co., Shiga, Japan) [22]. The mRNA levels of mitoferrin-2 expression were detected using QRT-PCR on a Light Cycler 480 (Roche Diagnostics, Basel, Switzerland) according to the manufacturer’s protocol. The primer set of mitoferrin-2 was: sense, 5′-GGAGCATTCCAGGAGACA-3′; antisense, 5′-GGGTGACCGCCTATTT-3′. Each sample was checked in triplicate, and parallel reactions were performed using primers to β-actin as an internal control. The data were analyzed using the Light Cycler 480 software.

### Western blot analysis

Cell extracts were prepared in ice-cold radioimmune precipitation assay lysis buffer (150 mM NaCl, 1 mM ethylene glycol tetraacetic acid (EGTA), 1% sodium deoxycholate, 1% Triton X-100, 0.1% SDS, 1% Nonidet P-40, 50 mM Tris-Cl, pH 7.4) supplemented with a mixture of protease inhibitors (Roche Diagnostics, Basel, Switzerland) and centrifuged. Equivalent amounts of protein determined by Bradford assay (Bio-Rad, California, USA) in sample buffer (Invitrogen) supplemented with 10% SDS and 10% β-mercaptoethanol were resolved on NuPAGE Tris-bis 12% polyacrylamide gels (Invitrogen, California, USA). Proteins were transferred to polyvinylidene difluoride (PVDF) membranes (EMD Millipore, armstadt, Germany) and probed with anti-Mfrn-2 (1:100) (Santa Cruz Biotechnology, Shanghai, China) and anti-β-actin (1:1,000) (Santa Cruz Biotechnology). Membranes were developed by two-color infrared fluorescence imaging system (LI-COR Biosciences, Nebraska, USA).

### ROS detection

Glioma cells were transfected with 40 pmol of siRNA duplex or negative groups and exposed to 5 μM As_2_O_3_ for 48 hours. Then cells were incubated with 10 μM 2′,7′-dichlorofluorescin diacetate (Sigma-Aldrich, St. Louis, MO, USA) for 30 minutes, after which they were washed. ROS generation was determined by FACS can flow cytometry (Becton-Dickinson Mountain View, CA, USA) using CellQuest software and fluorescent signals were displayed as histograms.

### MTT analysis

To investigate cell viability, U87MG and T98G cells were seeded in 96-well plates at a density of 5 × 10^3^ cells per well and stabilized for 24 hours. Glioma cells were transfected with 40 pmol of siRNA duplex or negative groups and exposed to 5 μM As_2_O_3_ for 48 hours. The cells were incubated with new culture medium containing MTT working solution (0.5mg/ml) for 4 hours at 37°C. The culture supernatant was removed from the wells, and dimethyl sulfoxide (DMSO) was added. The absorbance of each well was measured at a wavelength of 490 nm with a reader.

### Apoptosis

The apoptosis-inducing effects of As_2_O_3_ in U87MG and T98G cells were determined by flow cytometery using Annexin-V/FITC and propidium iodide. About 0.5 × 10^6^ cells were plated in each well of a 6-well plate and transfected with 40 pmol of siRNA duplex or negative groups and exposed to 5 μM As_2_O_3_ for 48 hours. Apoptosis was determined using human Annexin-V-FITC kit according to the manufacturer’s instructions and analyzed by flow cytometry. Tests were repeated in triplicate.

### Statistical analysis

Statistical analysis was performed by Student’s *t*-test. *P*-values of <0.05 were considered statistically significant. All values are expressed as mean ± SEM from three different experiments.

## Results

### Effects of As_2_O_3_ on cell viability in glioma

**Figure 1:**
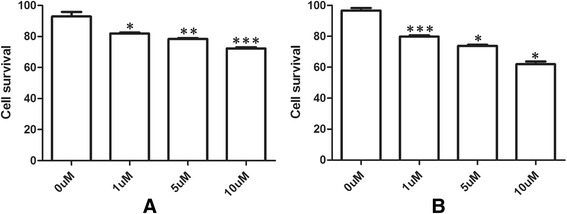
**After exposure of human glioma cell lines U87MG (A) and T98G (B) to As**_**2**_ **O**_**3**_ **at different doses for 48 hours, the cytotoxicity of As**_**2**_ **O**_**3**_ **was analyzed by MTT.** Student’s *t*-test was used for evaluation statistical significance (*P < 0.05).MTT, 3-(4,5-dimethylthiazol-2-yl)-2,5-diphenyltetrazolium bromide.

The sensitivity of U87MG and T98 G cells to As_2_O_3_ was first investigated by MTT. Figure 1 shows that the cytotoxic effect of a 48-hour treatment with As_2_O_3_ was dose-dependent, and 5 μM was chosen as the optimal concentration for As_2_O_3_ treatment in U87MG (Figure 1A) and T98G (Figure 1B).

### Induction of ROS and oxidative stress by As_2_O_3_

**Figure 2:**
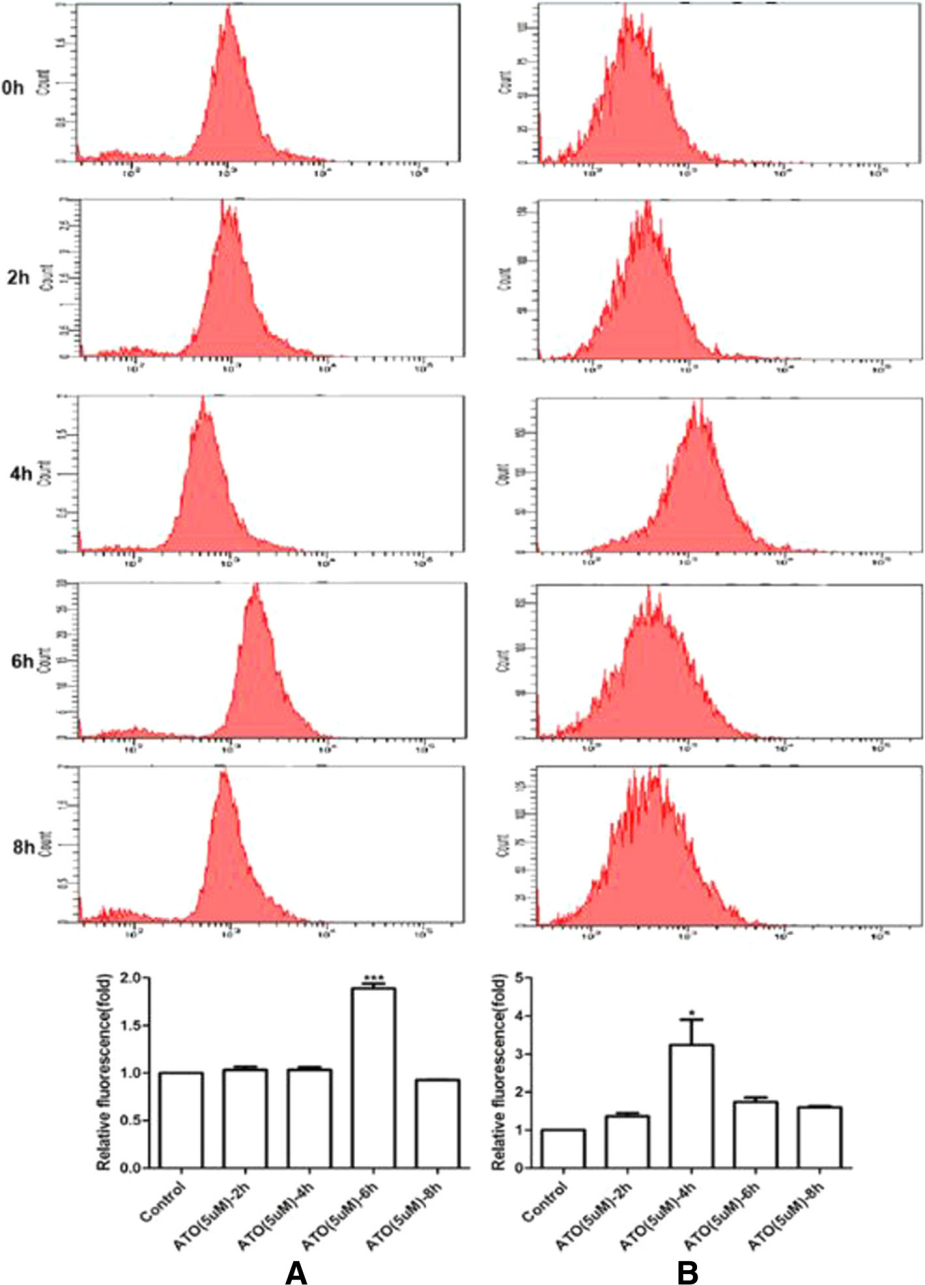
**After exposure of human glioma cell lines U87MG (A) and T98G (B) to As**_**2**_ **O**_**3**_ **at different times, the reactive oxygen species (ROS) production of As**_**2**_ **O**_**3**_ **was analyzed by flow cytometry.** Student’s *t*-test was used for evaluation statistical significance (*P < 0.05).

To explore the effect of As_2_O_3_ on oxidative stress production in glioma cells, the ROS production induced by As_2_O_3_ in U87MG and T98G was measured. At 2 hours, 4 hours, 6 hours or 8 hours after exposure to 5 μM As_2_O_3_, the intracellular ROS levels were increased and the peak production appeared at 6 hours and 4 hours respectively in U87MG (Figure 2A) and T98G cell lines (Figure 2B). Therefore, 6 hours and 4 hours were chosen times to monitor ROS production for the follow-up tests in U87MG and T98G, respectively.

### As_2_O_3_ enhanced the expression of mitoferrin-2 in glioma cells

**Figure 3:**
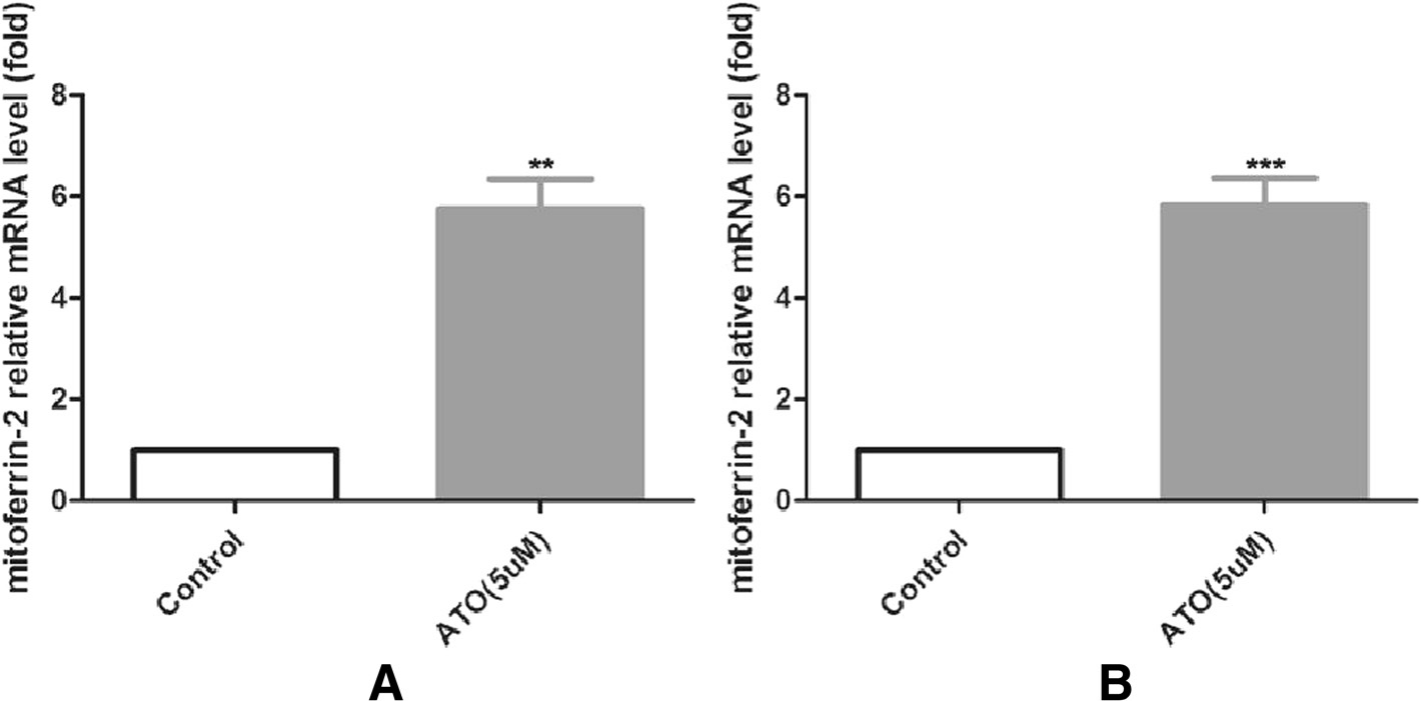
**Treatment with As**_**2**_ **O**_**3**_ **(5 μM, 4 8hours) significantly increased intracellular mitoferrin-2 expression in glioma cells U87MG (A) and T98G lines (B).** Data are presented as mean ± SEM for the separate experiments performed in duplicate. Student’s *t*-test was used to evaluate statistical significance (*P < 0.05).

**Figure 4:**
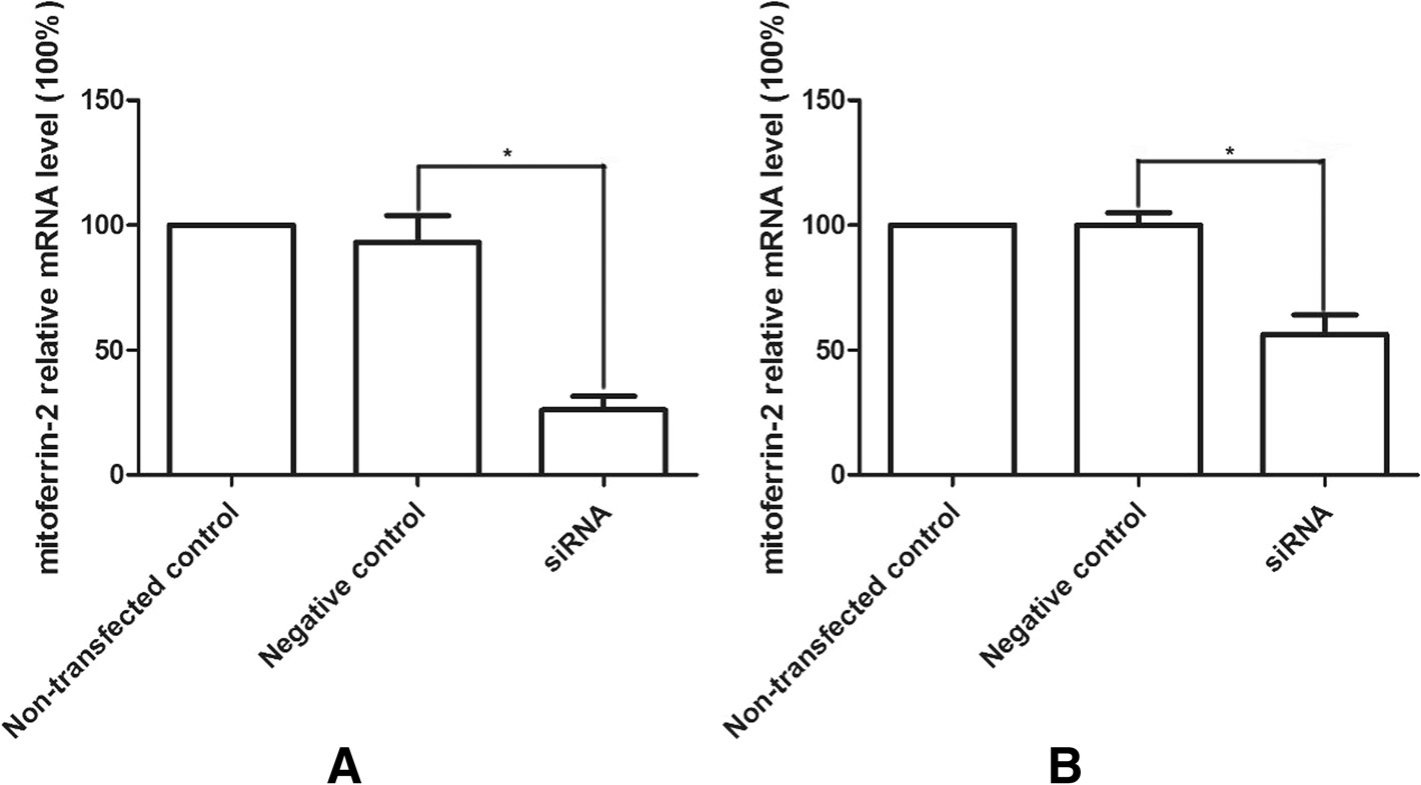
**Silencing mitoferrin-2 by siRNA for 48 hours was shown in U87MG (A) and T98G (B) lines.** Expression of mitoferrin-2 was decreased after using siRNA interferencing in both U87MG and T98G. Data are presented as mean ± SEM for the separate experiments performed in duplicate. Student’s *t*-test was used to evaluate statistical significance (*P < 0.05).

**Figure 5:**
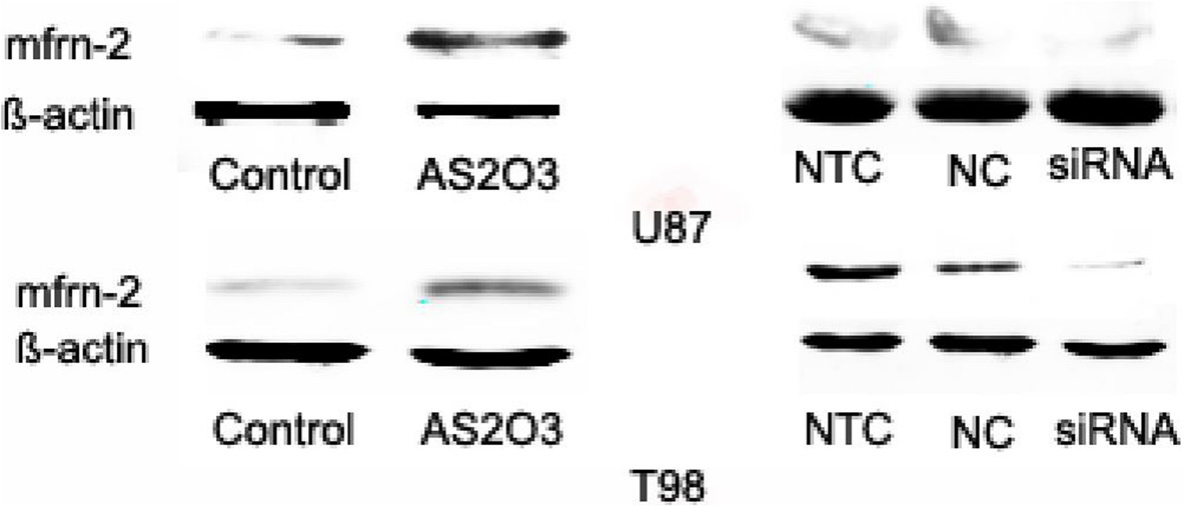
**The expressions of mitoferrin-2 in cell lysates were detected by Western blotting.** β-actin was used as an internal control. Pretreatment with As_2_O_3_ (5 μM, 48 hours) significantly increased intracellular mitoferrin-2 expression in glioma cell lines U87MG and T98G. Silencing mitoferrin-2 by siRNA for 48 hours was shown in both U87MG and T98G lines.

To explore the possible mechanism of As_2_O_3_-induced cell damage and ROS production, we measured the cellular mitoferrin-2 expression after pretreatment with As_2_O_3_ (5 μM) for 48 hours. Our data from QRT-PCR showed that the expression of mitoferrin-2 was increased about 4 to 5 folds in both U87MG (Figure 3A) and T98G lines (Figure 3B) after As_2_O_3_ pretreatment. The mitoferrin-2 expression after silencing was also measured by QRT-PCR. As shown in Figure 3, the expression rate of mitoferrin-2 mRNA decreased about 67.4% and 43.4% respectively in U87MG (Figure 4A) and T98G lines (Figure 4B) transfected with mitoferrin-2 siRNA as compared to negative control siRNA oligonucleotides. Mitoferrin-2 expression measured by Western blot analysis (Figure 5) produced the same results as with QRT-PCR.

### ROS production and apoptosis in As_2_O_3_-treated glioma cells decreased after silencing the mitoferrin-2 expression

**Figure 6:**
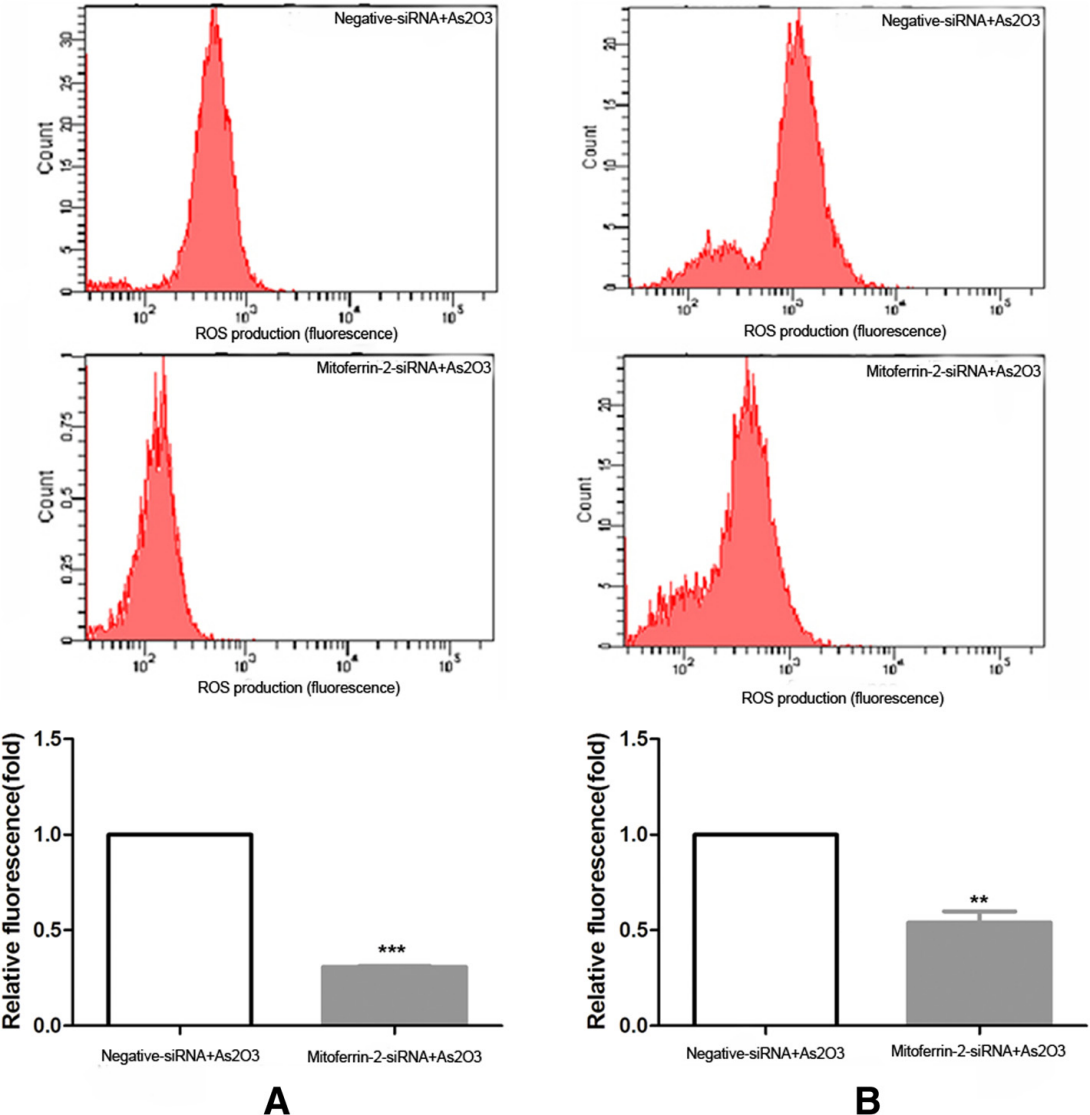
**Reactive oxygen species (ROS) production was measured by flow cytometry in human glioma cell lines U87MG (A) and T98G (B).** The ROS production in the silenced mitoferrin-2 group following pretreatment with As_2_O_3_ was decreased compared with the As_2_O_3_ (5 μM) group. Fluorescence signals represent ROS production. Data are presented as mean ± SEM for the separate experiments performed in duplicate. Student’s *t*-test was used to evaluate statistical significance (*P < 0.05).

**Figure 7:**
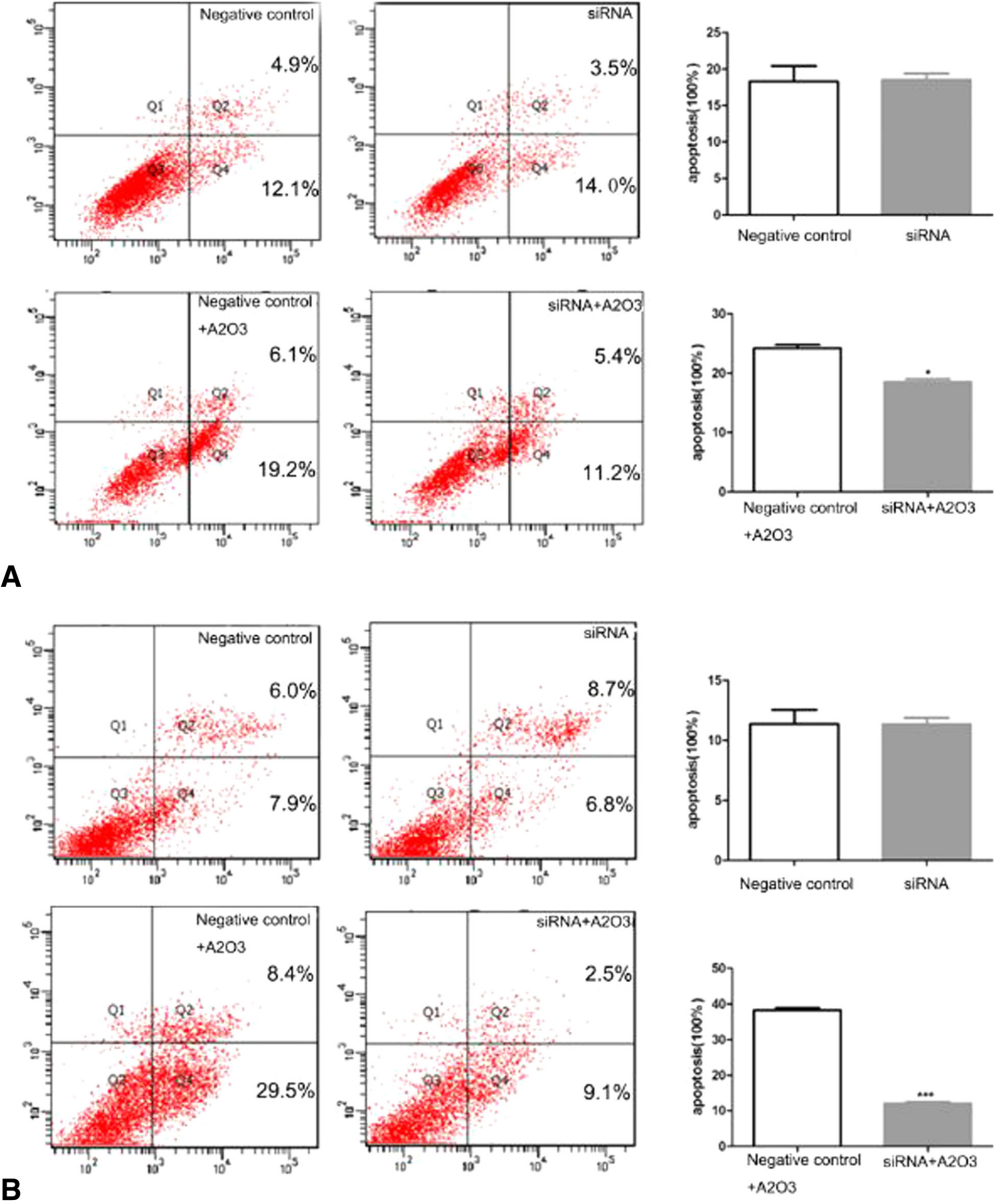
**Apoptosis in As**_**2**_ **O**_**3**_ **-pretreated human glioma cells was measured by flow cytometry.** The apoptosis rate was decreased in the silenced mitoferrin-2 group (48 hours) following pretreatment with As_2_O_3_ (5 μM, 48 hours) compared with the As_2_O_3_ (5 μM, 48 hours) group in both human glioma cell lines U87MG **(A)** and T98G **(B)**. Student’s *t*-test was used to evaluate statistical significance (*P < 0.05).

**Figure 8:**
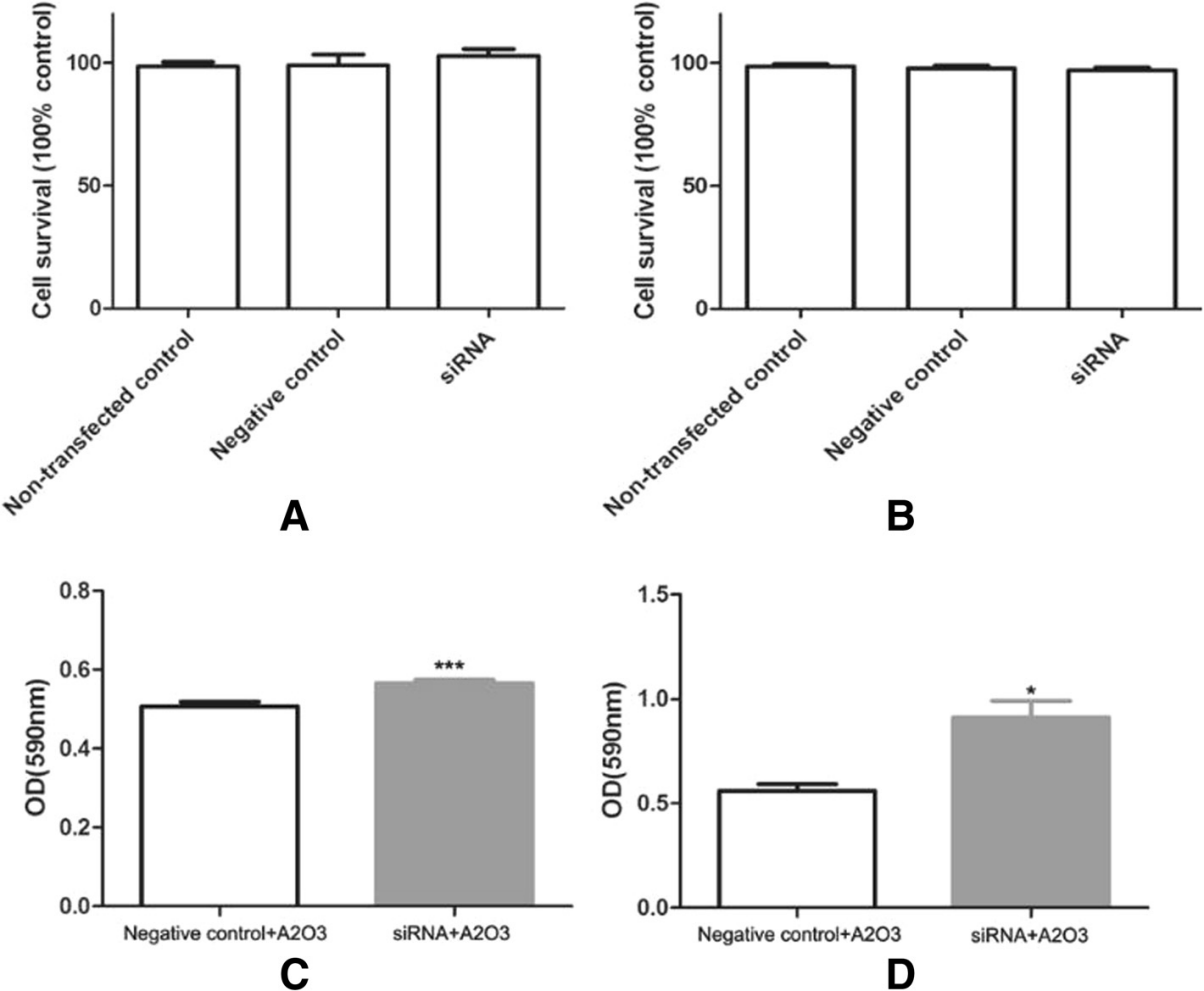
**Proliferation in As**_**2**_ **O**_**3**_ **-pretreated human glioma cells was measured by MTT.** Proliferation rates were not changed in either human glioma cell lines U87MG **(A)** or T98G **(B)** after silencing mitoferrin-2 for 48 hours. Proliferation rates were increased in the silenced mitoferrin-2 group following pretreatment with As_2_O_3_ for 48 hours compared with the As_2_O_3_ (5 μM) group in both human glioma cell lines U87MG **(C)** and T98G lines **(D)**. Student’s *t*-test was used to evaluate statistical significance (*P < 0.05). MTT, 3-(4,5-dimethylthiazol-2-yl)-2,5-diphenyltetrazolium bromide.

To confirm the role of mitoferrin-2 transporter in As_2_O_3_ treatment of glioma cells, we modulated mitoferrin-2 expression in U87MG and T98G by using siRNA interferencing. For those cells pretreated with As_2_O_3_ (5 μM), the ROS production (Figure 6), apoptosis (Figure 7) and cell death rates (Figure 8) were decreased after silencing the mitoferrin-2 transporter. siRNA-mediated inhibition of As_2_O_3_-induced apoptosis is greater in T98MG compared with U78MG (Figure 7). These data indicated mitoferrin-2 silencing strongly protected against ROS production induced by As_2_O_3_.

## Discussion

As_2_O_3_ has achieved some efficacy in treating glioma [23–25] although the mechanism remains unclear. In our study, the results indicated that mitoferrin-2, by mediating ferrous transport across the mitochondrial inner membrane, plays a significant role in As_2_O_3_-induced glioma cell death. Our findings showed that the expression level of mitoferrin-2 was increased about 4 to 5 folds in both U87MG and T98G after pretreatment with As_2_O_3_ (5 μM) after 48 hours. Next, to determine whether mitoferrin-2 mediates mitochondrial ROS production or apoptosis in As_2_O_3_-induced glioma cell damage, *mitoferrin-2* gene expression was silenced by siRNA in glioma cells and exposed to As_2_O_3_ for 48 hours. Our data showed that ROS production and apoptosis in low mitoferrin-2 expression groups were reduced compared to negative control groups in As_2_O_3_ pretreated glioma cells. Additionally, we firstly demonstrated that down-regulation of mitoferrin-2 expression affects As_2_O_3_-induced cell toxicity in human glioma cell lines U87MG and T98G. As_2_O_3_-induced cytotoxicity was reduced after silencing mitoferrin-2. Therefore, we assume that mitoferrin-2 transporter may partly participate in As_2_O_3_-induced cytotoxicity through promotion of ROS production.

Human glioma treatment is especially challenging because current treatments, such as chemical therapy, drastically affect patient quality of life. As_2_O_3_ has been successfully used for gliomas in a number of clinical trials and experiments because of its significant anticancer property [1,2]. In previous researches, As_2_O_3_ (3 to 10 μM) could also induce apoptosis in cultured bone marrow mesenchymal stem cells [26,27]. Additionally, treatment with high concentrations of As_2_O_3_ (30, 60, and 90 μM) caused primary cardiomyocyte apoptosis in a dose- and time-dependent manner [28]. Higher concentrations of As_2_O_3_ (4 to 32 μM) obviously reduced smooth muscle cell viability in a concentration-dependent manner [29]. One clinical study has indicated that the plasma levels of arsenic are 5.54 to 7.30 μM in acute promyelocytic leukemia patents treated with As_2_O_3_ for detection of ROS activity [30]. In the present study, we found that treatment with As_2_O_3_ (1 to 10 μM) in glioma cell lines significantly decreases cell viability and increases cell apoptosis.

Further, As_2_O_3_ is a mitochondrial toxin causing ROS generation [31], mitochondrial transmembrane potential disappearance, and cytochrome C release into the cytoplasm. These phenomena could be the cause of apoptosis. Therefore, we presume that As_2_O_3_ induces apoptosis in tumor cells possibly by affecting mitochondrial function [22,32–38]. Our data showed that highest levels of As_2_O_3_-induced ROS production were after 4 hours and 6 hours in U87MG and T98G cell lines respectively. Additionally, previous researches demonstrated that intracellular ROS mediates multiple cellular responses, including cell cycle progression [39], cell differentiation [40], and apoptotic cell death [41]. As we know, ROS is associated with the collapse of matrix metal proteinases (MMP) and the subsequent oxidative damage to the mitochondrial membranes, leading to disruption of MMP, cytochrome release, and apoptosis [42]. Therefore, ROS plays an important role in the antitumor effect of As_2_O_3._

Previous researches have also reported that iron is associated with ROS production in some diseases [7]. Because of its redox properties, the transient existence of ferrous in mitochondria can catalyze the production of ROS that can be highly toxic through the Fenton reaction [10]. However, it has long been known that, when in excess, ROS are among the major determinants of toxicity in cells and organisms. Therefore, mediated mitochondrial iron accumulation may affect As_2_O_3_-induced ROS production in human glioma.

Mitoferrin-2-dependent mitochondrial iron uptake acts synergistically to induce photodynamic therapy (PDT)-mediated and iron-dependent mitochondrial dysfunction and subsequent cancer cell killing has been reported [20]. Therefore, whether mitoferrin-2 can affect the antitumor effect of As_2_O_3_ should be further explored. In our experiment, mitoferrin-2 mRNA expression was up-regulated in glioma cells after pretreatment with As_2_O_3_. Thus, mitoferrin-2 may participate in As_2_O_3_ treatment. Additionally, previous researches have reported that up-regulated expression of mitoferrin in yeast and mouse models of Friedreich’s ataxia suggested that it is associated with the pathogenesis of diseases with mitochondrial iron accumulation [43]. Down-regulating the expression of mitoferrin-2 or blocking the iron import channel by some approaches should alleviate the symptoms of patients by preventing iron accumulation [44]. Therefore, we assume that mitoferrin-2 may regulate mitochondrial iron accumulation and participate in As_2_O_3_-induced ROS production and cell damage.

In order to further explore whether mitoferrin-2 plays a crucial role in As_2_O_3_-induced cytotoxicity, we measured ROS production in both down-regulated mitoferrin-2 expression groups and negative groups in glioma cells pretreated with same-concentration As_2_O_3_. Our data showed that ROS production in low mitoferrin-2 expression groups was reduced compared to negative groups in glioma cells pretreated with As_2_O_3_. This result suggests that mitoferrin-2 can regulate As_2_O_3_-induced ROS production in glioma cells. Additionally, mitoferrin-2 has been demonstrated to increase the iron transmitted into mitochondria and to be related to ROS production in PDT [20]. The predominant proportion of ROS production occurs inside mitochondria, leading to mitochondrial permeability transition and cell death [15]. Therefore, overall these findings suggest that mitoferrin-2 has anapoptotic-promotingrole in As_2_O_3_-induced glioma apoptosis and cell damage.

## Conclusion

In summary, we have demonstrated for the first time that mitoferrin-2 transporter plays an important role in the antitumor effect induced by As_2_O_3_ in human glioma cells. These findings suggest that mitochondrial mitoferrin-2 transporter may be an important potential regulator in As_2_O_3_-induced glioma cell death and further understanding mechanisms in both the therapeutic activities and the toxicities of arsenic. Additionally, together with the multiple molecular targets affected by arsenic, suggests the potential for additive or even synergistic effects when arsenic is administered with other cytostatic or cytotoxic agents. Future studies are needed to investigate whether mitoferrin-2 can take part in As_2_O_3_ treatment in human glioma *in vivo* and in the clinical setting.

## Abbreviations

As_2_O_3_: Arsenic trioxide
ROS: Reactive oxygen species
MTT: 3-(4,5-dimethylthiazol-2-yl)-2,5-diphenyltetrazolium bromide
ISC: Iron-sulfur cluster
DMSO: Dimethylsulfoxide
EDTA: Ethylenediaminetetraacetic acid
QRT-PCR: Quantitative real- time -PCR
EGTA: Ethylene glycol tetraacetic acid
PVDF: Polyvinylidene difluoride
MMP: Matrix metal proteinases
PDT: Photodynamic therapy
